# Some Problems of To-Day

**Published:** 1918-04-27

**Authors:** 


### SOME PROBLEMS OF TO-DAY.

By A SOCIAL WORKER.

### THE UNMARRIED MOTHER.

THE magazine which began last year, under the attractive title of *Maternity and Child Welfare,* * has no doubt received the attention it deserves from readers interested in these pressing questions. Perhaps others have also been attracted by Mr. Byam Shaw's design on the cover and his cartoons inside. The fine drawing in one number might with advantage be reprinted. It represents a curly-headed laddie set in the pillory for the crime of being illegitimate, while above him stands Justice, tall, blindfolded, carrying her scales, and *Why?* is inscribed underneath. Attention, however, must be held by continued interest, and so far the high level of thought and diction promises well for the spreading of interest in this great subject. All over the country experiments in child welfare work have been many and varied, and variety is greatly to be desired. But there comes a point when inter-communication is very necessary if the fruits of experience are to be usefully shared. The establishment of an Inquiry Bureau by the Child Welfare Council, representing some seventy bodies interested in the children, offers help in many directions. It is good to know that this office is being used by the distressed unmarried mother who seeks a home for herself and her babe. A specially commendable point marking the articles in this magazine and the resulting correspondence is the frank statement of difficulties and failures as well as of progress. We are far indeed still from being able to lay down rails on which help may be conveyed from the mines of public or private goodwill to those who need. In the case of the unwanted child emotion cannot be too deeply touched or the best brains too busily employed to remedy his undeserved griefs and their damaging effect upon family and national life, and the organised discussion started in this magazine is an excellent step.

### PROGRESS IN THE LAST HALF CENTURY.

It is difficult to think ourselves back to the late 'seventies of last century, when, as Mr. Parr, of the N.S.P.C.C., has lately recalled, some awakened interest was frowned upon by many good people as encouragement to vice. Probably few men understood that the unwelcomed child is very seldom that of the professional prostitute; certainly no one realised how seriously the problem of feeble-mindedness was entangled with the obvious question. Then, as now, it was true that girl or man seldom cast a thought forward to the happiness or the cares of parenthood, and probably more true than now that many highly-placed persons did not sincerely desire to abolish the social evil. Let us be thankful that efforts persevered in have decreased such births from 53 to 7 in 1,000. The gain is great, even if allowance has to be made for more care to avoid "trouble." But we cannot rest content that all is well while these poor babes die two or three times more often than the fathered, especially with our present knowledge that a high death-rate means a high rate of damaged survivors. We gather indications of the conditions from such facts as that in 1910 deaths from syphilis were eight times higher among the illegitimate than among others. How many lived to carry on the contamination?

The plain question is, How are we to save these little ones, already deprived of half their birthright, from further undeserved suffering? Let us not forget that all influences which have raised the moral tone have further penalised them. How, again, can we mould them into useful citizens rather than wastrels burdening the community? How shall we secure that the father faces his responsibility, that the mother exercises her motherhood, and that in all possible and fit cases marriage and a real home, best of remedies, may be applied, and no girl ever again driven to despair and perhaps self-murder or child-murder for want of help? How, at the same time, shall we do these things without hindering any influences which should stop the trouble at its source? The fear of indifference in girls as the result of removal of all penalties is not an unreal one, though we may well feel that nothing in our power to do can really make up for what should have been.

### WHAT IS NEEDED.

We must certainly begin before birth. What effect on the child must not the pre-natal influences have if its mother is wild with passion, or half-crazed by anguish and dread, or even cynically indifferent? On the other hand, she may become responsive as never before to uplifting suggestions. Then how shall mother and child come safely through the crisis of birth? Official midwives are frequently forbidden to attend unmarried cases, though the rule may often be evaded by sending for one when the girl is actually in labour. Mr. Parr proposes that the girl should register as an expectant mother, at the same time naming the father. That the fear of blackmail is negligible is shown by the experience of Norway under the 1915 law, under which proceedings were taken in eighteen months, and the number of cases questioned was only one in 1,000. Under our present unsatisfactory system it was shown that in 1911 only one woman in four made application to recover maintenance, and only one in five of these obtained the necessary order. It was stated during Baby Week by Mrs. Booth that as often as not the girl only knew the man by some nickname, in the case of a soldier very seldom his number or even his regiment. The order itself does not automatically secure payment. Nor, if the father is reached and does his duty, has he any security that the money will be spent, or properly spent, upon the child. Then at present our law lays upon the mother responsibility for maintaining the child till sixteen. But if she must work to keep it, how will she afford some 8s. a week for its keep, besides its clothing and her own expenses?

### SUPERVISION AND VISITATION.

And what of the foster-mother? All experience shows that these children need more careful training in all ways than the average lawful child. But much evidence goes to show that the child received for care, or adopted, needs careful watching. In spite of one vigorous letter setting forth the possible risk of forming a "marked class" by regular visitation, the arguments of this, as probably of all other discussions, go to show that what is needed is to provide a woman agent of the local court to register and watch over the mother, and make the child a ward of the court, or of the town mayor, or of a small committee of the Council. Communication with the father would then be made officially. If the Bill which Lord Lytton has lately told us is being drafted on the lines of Judge Neil's American system of mothers' pensions becomes law, the destitute, unmarried mother might—as in some, though not all, American States—be appointed

* Price 6d. monthly (John Ball, Great Titchfield Street, W.).

**SOME PROBLEMS OF TO-DAY**—*(continued)*.

caretaker of her own child. In any case, supervision and visitation are very necessary indeed, and not least perhaps where the mother marries and has other children, especially if she marries a widower. One of the most poignant cases known to the present writer is that of a little girl who is a speckled bird among a large double family. The results of a very little attention, given partly through the school teachers, have become most evident in the child's much improved state, and the sense of responsibility driven home to her mother. The school help is valuable, for no one can desire that the number of visitors already entering the homes of working people should be increased. Their patience and courtesy under what would be to more educated people a great infliction are remarkable. But one wholesome result of a Ministry of Health may be that the visitors will be more competent, better distributed, and clear as to their mission, so as to be worthy of the confidence so freely given them.

